# Angiopoietin-2 Is Associated with Albuminuria and Microinflammation in Chronic Kidney Disease

**DOI:** 10.1371/journal.pone.0054668

**Published:** 2013-03-01

**Authors:** Fan-Chi Chang, Tai-Shuan Lai, Chih-Kang Chiang, Yung-Ming Chen, Ming-Shiou Wu, Tzong-Shinn Chu, Kwan-Dun Wu, Shuei-Liong Lin

**Affiliations:** 1 Renal Division, Department of Medicine, National Taiwan University Hospital, Taipei, Taiwan; 2 Department of Internal Medicine, National Taiwan University Hospital Chu-Tung Branch, Hsin-Chu County, Taiwan; 3 Graduate Institutes of Physiology, College of Medicine, National Taiwan University, Taipei, Taiwan; 4 Department of Internal Medicine, National Taiwan University Hospital Bei-Hu Branch, Taipei, Taiwan; University of Washington, United States of America

## Abstract

Although cardiovascular disease (CVD) is the leading cause of mortality in patients with chronic kidney disease (CKD), the pathophysiology is not thoroughly understood. Given that elevated albuminuria or circulating angiopoietin-2 associates with CVD and mortality in CKD patients, we were intrigued by the relationship between albuminuria and angiopoietin-2. A total of 416 patients with CKD stages 3 to 5 were stratified by urine albumin-creatinine ratio as normoalbuminuria (<30 mg/g), microalbuminuria (30–300 mg/g), or macroalbuminuria (>300 mg/g). The levels of plasma angiopoietin-2 and vascular endothelial growth factor (VEGF) increased, and soluble Tie-2 decreased in the subgroups of albuminuria; whereas angiopoietin-1 did not change. Linear regression showed a positive correlation between urine albumin-creatinine ratio (ACR) and plasma angiopoietin-2 (correlation coefficient *r* = 0.301, 95% confidence interval 0.211–0.386, *P*<0.0001), but not between ACR and VEGF or soluble Tie-2. Multivariate linear regression analysis showed that plasma angiopoietin-2 was independently associated with ACR (*P* = 0.025). Furthermore, plasma angiopoietin-2 was positively correlated with high sensitive C-reactive protein (*r = *0.114, 95% confidence interval 0.018–0.208, *P* = 0.020). In conclusion, plasma angiopoietin-2 was associated with albuminuria and markers of systemic microinflammation in CKD patients. Although previous evidence has shown that angiopoietin-2 destabilizes vasculature and induces inflammation in different scenarios, further study will be required to delineate the role of angiopoietin-2 in albuminuria and microinflammation in CKD patients.

## Introduction

It is well established that chronic kidney disease (CKD) is an independent risk factor of cardiovascular disease (CVD). Patients with CKD are more likely to die of CVD than to enter dialysis. High prevalence of traditional Framingham risk factors in CKD patients undoubtedly leads to high cardiovascular events. Nevertheless, the non-traditional risk factors more precisely elucidate the CKD-related elements.

Albuminuria is one of the manifestations in renal glomerular disease. Moreover, it is also indicative of endothelial damage and vascular disease in diverse populations. Evidence has shown that albuminuria is an independent predictor of cardiovascular events in both CKD and non-CKD patients [1], [2], [3], [4], [5], [6]. Moreover, systemic microinflammation also infers the increased cardiovascular morbidity and mortality [7], [8], [9]. Although albuminuria and microinflammation explain the complex interplay in CKD, the effectors mediating the cross talk between CKD and CVD are still not confirmative.

Angiopoietin-1 (Ang-1) and angiopoietin-2 (Ang-2) are ligands of the Tie-2 receptor, the second class of vascular specific receptor tyrosine kinases in vascular development. The Ang/Tie-2 system regulates the quiescent and activated endothelial phenotype in a unique and nonredundant fashion [10]. Ang-1-mediated Tie-2 activation is required to maintain the quiescent resting endothelium [11], [12]. Ang-1 functions are antagonized by Ang-2. Ang-2 destabilizes the quiescent endothelium and primes it to respond to exogenous stimuli, thereby modulate the activities of inflammatory (tumor necrosis factor-α, TNF-α) and angiogenic (vascular endothelial growth factor, VEGF) cytokines [10], [13]. Concomitant occurrence of Ang-2 and other stimuli, such as TNF-α or VEGF, will promote endothelial cell proliferation, facilitate angiogenesis and induce inflammation. In the absence of VEGF, the endothelium switches back to the resting state, resulting in endothelial cell apoptosis and vascular regression. Elevated plasma Ang-2 has been shown in diseases with systemic inflammation including diabetes mellitus, hypertension, congestive heart failure, acute coronary syndrome, peripheral artery disease, critical illness, CKD and end-stage renal disease (ESRD) [14], [15], [16], [17], [18], [19], [20]. Notably, previous studies have shown that circulating Ang-2 levels are associated with CVD and predict the long-term mortality in CKD patients [20], [21], [22].

Given that elevated albuminuria and circulating Ang-2 predict CVD and mortality in CKD, we were intrigued by the relationship between albuminuria and Ang-2. In this cross-sectional study, we aimed to define the relationship between plasma Ang-2 levels and albuminuria in patients with CKD stage 3 to 5.

## Materials and Methods

### Study Population

A cross-sectional study was performed in a single tertiary medical center in Taiwan from December 2006 to December 2007. Patients aged more than 18 years old from the outpatient clinic with CKD were eligible for inclusion. CKD was defined by either kidney damage or GFR criteria for at least 3 months [23]. We used the four-variable equation of the Modification of Diet in Renal Disease (MDRD) Study to estimate the GFR (eGFR) [24]. The urine albumin-creatinine ratio (ACR) was measured by dividing the urine albumin to creatinine concentration. Proteinuria based on ACR was defined as normoalbuminuria (<30 mg/g), microalbuminuria (30–300 mg/g), or macroalbuminuria (>300 mg/g). We excluded patients with current infection, malignancy, pregnancy, who had already received a kidney transplant or were receiving maintenance dialysis. All patients were maintained on their regular medication. The study was approved by the institutional review board of National Taiwan University Hospital (201105023RC) and registered. Written informed consent was obtained from all participants.

### Ascertainment of Covariates

Patients were classified as hypertensive if systolic blood pressure ≥140 mmHg, diastolic blood pressure ≥90 mmHg, or with antihypertensive drugs use. Diabetes was defined by history and blood glucose values (using the American Diabetes Association criteria), oral hypoglycemic medication, or insulin use. The clinical definition of dyslipidemia was fasting total cholesterol ≥200 mg/dL, low-density lipoprotein ≥130 mg/dL, triglyceride ≥200 mg/dL, or lipid-lowering medication.

### Blood and Urine Samplings

Fasting blood samples were collected in the morning. Blood samples were taken by antecubital venopuncture in the sitting position after resting for more than 5 minutes. The blood samples were collected into EDTA vacutainers and placed on ice immediately. Within 30 minutes of collection, samples were centrifuged at 3000 rpm (1000 g) and 4°C for 15 minutes. After overnight fasting, a random morning urine sample was collected for measurement of creatinine and albumin. The additional first-void specimens were collected after 3 months to confirm the presence of albuminuria. Plasma and urine were kept frozen in aliquots. Complete blood cell count and all biochemical analyses were performed in the Department of Laboratory Medicine, National Taiwan University Hospital.

### Enzyme-linked Immunosorbent Assay

Plasma Ang-1, Ang-2, VEGF and soluble Tie-2 (sTie-2) were measured in duplicate using commercial enzyme-linked immunosorbent assays (R&D System) according to the instructions of the manufacturer. The sensitivities of Ang-1, Ang-2, VEGF, and sTie-2 assays were 1.36, 1.20, 1.61, and 1.00 pg/mL, respectively. Intraassay coefficients of variation of Ang-1, Ang-2, VEGF, and sTie-2 were 2.1%, 1.3%, 1.6%, and 0.8%, respectively. Interassay coefficients of variance of Ang-1, Ang-2, VEGF, and sTie-2 were 1.8%, 2.0%, 2.1%, and 9.3%, respectively.

### Statistical Analysis

Since the continuous baseline variables, including angiotrophic growth factors, were not normally distributed, differences between these variables among albuminuric status were analyzed using Kruskall-Wallis test. Differences in categorical variables were compared using the chi square test. To examine the correlations between Ang-2 and albumin, uric acid, high sensitive C-reactive protein (hsCRP) and ACR, Ang-2 was log-transformed. Pearson correlation coefficients were computed.

The Shapiro-Wilk test was used to test normality. Logarithmic transformation was made for variables when needed. A linear regression model was used to assess the relationship between Ang-2 and albuminuria. We first computed the univariate regression analysis and then generated several multivariable models. First, we adjusted for age and gender in model 1. Second, we further adjusted for all traditional and nontraditional risk factors for albuminuria, including diabetes, hypertension, dyslipidemia, mean brachial systolic blood pressure, eGFR levels, calcium phosphate product, hemoglobin, hsCRP, and medications in use. Estimated glomerular filtration rate was treated as a continuous variable. Medications included angiotensin converting enzyme (ACE) inhibitors or angiotensin II receptor blockers (ARBs), statins, calcium channel blocker, β-blocker, pentoxifylline. Each of them was treated as a categorical variable. Finally, we used the stepwise regression method to select variables. All statistical analyses were performed with STATA, version 9 (Stata Corp., College Station, Texas, USA).

## Results

### Clinical Characteristics of Patients

Total 416 participants with CKD stage 3 to 5 (average age 63 yr; 63.5% men) were analyzed (Table 1). We stratified patients into three groups according to the urine ACR level. Of all patients, 51.4% have macroalbuminuria (n = 214), 31.7% microalbuminuria (n = 132) and 16.8% are normoalbuminuria (n = 70). There is significant elevation of serum creatinine, phosphate and intact parathyroid hormone in patients with macroalbuminuria (*P*<0.0001). Likewise, anemia is more severe in the microalbuminuria and macroalbuminuria group (*P* = 0.001 and *P*<0.0001, respectively). Elevated uric acid and decreased albumin level were evident in patients with macroalbuminuria (*P* = 0.001 and *P*<0.0001) but not microalbuminuria group. Inflammatory biomarkers, including hsCRP and ferritin, did not vary with albuminuria.

**Table 1:** Clinical characteristics of the patients^a^.

Albuminuria^b^	All (n = 416)	normoalbuminuria (n = 70)	microalbuminuria (n = 132)	macroalbuminuria (n = 214)	*P* c
Age (yr)	63.0 (54.0–71.0)	68.0 (56.0–73.0)	65.5 (55.0–71.0)	59.0 (50.8–71.0)**	0.005
Men [%, (n)]	63.5 (264)	78.6 (55)	62.1 (82)*	59.3 (127)*	0.014
Diabetes [%, (n)]	39.2 (163)	34.3 (24)	26.5 (35)	48.6 (104)*	<0.0001
Hypertension [%, (n)]	86.8 (361)	87.1 (61)	81.1 (107)	90.2 (193)	0.051
Smoker [%, (n)]	11.1 (46)	10.0 (7)	9.8 (13)	12.1 (26)	0.765
Dyslipidemia [%, (n)]	30.0 (125)	24.3 (17)	28.0 (37)	33.2 (71)	0.307
BMI (kg/m^2^)	24.4 (22.0–27.0)	24.7 (22.5–26.7)	24.0 (21.2–27.1)	24.4 (22.2–27.1)	0.334
eGFR (mL/min/1.73 m^2^)	27.3 (14.4–41.0)	42.0 (34.4–51.5)	29.5 (18.0–41.7)**	20.3 (10.6–34.1)**	<0.0001
Creatinine (mg/dL)	2.3 (1.7–4.2)	1.6 (1.4–2.0)	2.1 (1.6–3.6)**	3.0 (2.0–5.1)**	<0.0001
Albumin (g/dL)	4.5 (4.2–4.7)	4.6 (4.5–4.8)	4.6 (4.3–4.8)*	4.4 (4.1–4.6)**	<0.0001
Calcium (mmol/L)	2.32 (2.26–2.42)	2.38 (2.30–2.48)	2.34 (2.27–2.42)*	2.30 (2.23–2.39)**	<0.0001
Phosphate (mg/dL)	3.8 (3.3–4.5)	3.3 (3.0–3.6)	3.7 (3.3–4.2)**	4.2 (3.6–4.9)**	<0.0001
Urine albumin-creatinine ratio(mg/g)	332.8 (59.4–794.5)	14.7 (8.6–19.6)	105.6 (58.6–178.3)**	762.5 (537.2–1205.8)**	<0.0001
Hemoglobin (g/dL)	11.5 (9.9–13.5)	13.3 (11.4–14.4)	11.9 (9.7–13.6)*	11.1 (9.4–12.9)**	<0.0001
hsCRP (mg/dL)	0.120 (0.063–0.260)	0.110 (0.070–0.313)	0.130 (0.070–0.280)	0.120 (0.060–0.210)	0.392
iPTH	92.1 (51.2–176.0)	64.0 (41.9–86.6)	84.3 (50.1–147.8)*	119.0 (58.0–273.3)**	<0.0001
Uric acid (mg/dL)	8.2 (7.1–9.4)	7.5 (6.3–8.9)	7.8 (6.8–9.2)	8.5 (7.5–9.5)*	<0.0001
Ferritin	173.0 (103.0–282.8)	183.0 (104.3–324.5)	157.0 (100.3–268.3)	185.0 (104.5–283.8)	0.478

Note:

a Continuous and categorical variables were expressed as median (interquartile range) and percentage (number) respectively.

b Patients were stratified based on albuminuria (urine albumin-creatinine ratio) as normoalbuminuria (<30 mg/g), microalbuminuria (30–300 mg/g), macroalbuminuria (>300 mg/g).

c Chi-Square test in categorical variables, Kruskall-Wallis test in continuous variables.

* P<0.05 compared with normoalbuminuria group (Chi-Square test in categorical variables, Mann-Whitney U test in continuous variables).

** P<0.0001 compared with normoalbuminuria group (Chi-Square test in categorical variables, Mann-Whitney U test in continuous variables).

Abbreviations: BMI, body mass index; eGFR, estimated glomerular filtration rate; Calcium phosphate product, Calcium x Phosphate x 4; hsCRP, high sensitivity C-reactive protein; iPTH, intact parathyroid hormone.

### Plasma Levels of Angiotrophic Growth Factors

**Figure 1:**
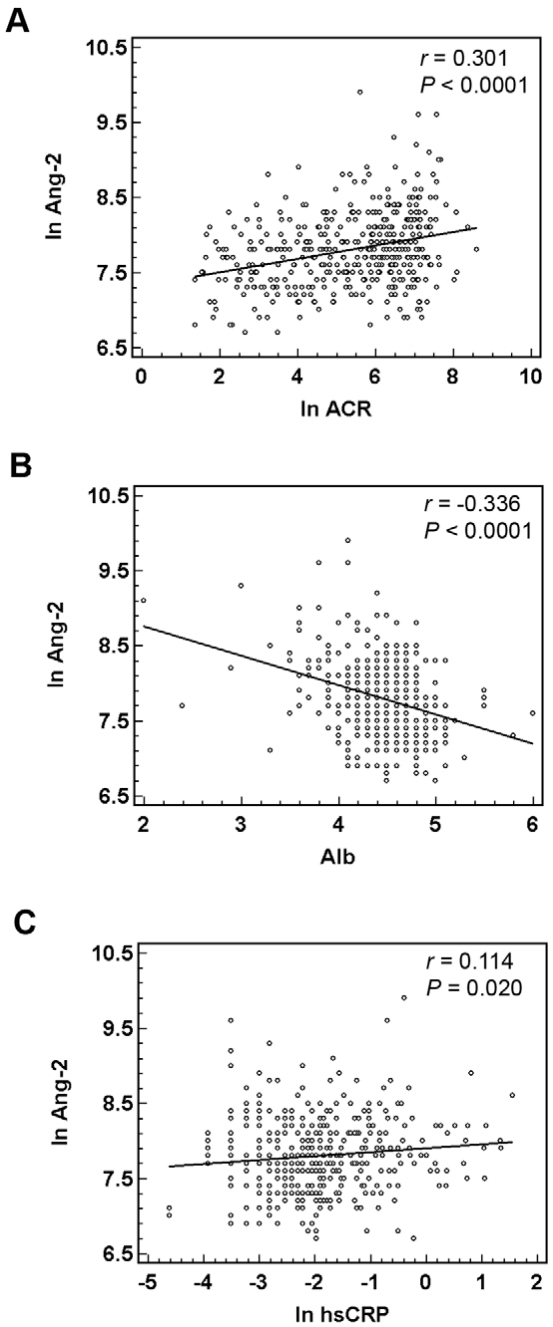
Plasma angiopoietin-2 was correlated with malnutrition, inflammation, and albuminuria. Univariate regression analysis showed the linear correlation of plasma angiopoietin-2 (Ang-2) with urine albumin-creatinine ratio (ACR) (A), high sensitive C-reactive protein (hsCRP) (B), and serum albumin (C). The levels of plasma Ang-2, ACR and hsCRP were expressed as natural logarithm (ln); *r*, Pearson correlation coefficient.

Although there was no difference in the plasma levels of Ang-1 in the subgroups of albuminuria (*P* = 0.355, Table 2), plasma levels of Ang-2 were higher in the subgroups (*P*<0.0001, Table 2) and showed a positive correlation with ACR (correlation coefficient *r* = 0.301, 95% CI 0.211–0.386, *P*<0.0001, Fig. 1A). Although the plasma levels of VEGF were also higher in the subgroups of albuminuria (*P* = 0.001, Table 2), linear regression revealed no correlation between ACR and VEGF. Likewise, even though the plasma levels of sTie-2 were lower in the subgroups of albuminuria (*P* = 0.011, Table 2), there was no linear correlation with ACR.

**Table 2:** Plasma levels of angiotrophic growth factors.

Albuminuria	All (n = 416)	normoalbuminuria(n = 70)	microalbuminuria(n = 132)	macroalbuminuria(n = 214)	*P*
Ang-1 (pg/mL)	6636.3 (2850.0–11690.4)	6784.8 (2286.6–12698.2)	5707.3 (2779.4–10754.1)	6821.9 (2905.9–13082.5)	0.355
Ang-2 (pg/mL)	2355.1 (1726.3–3287.1)	1863.2 (1434.5–2448.7)	2252.3 (1679.7–2964.6)*	2580.2 (2000.7–3775.6)**	<0.0001
VEGF (pg/mL)	126.5 (69.7–215.0)	92.7 (33.9–174.9)	105.6 (60.7–195.3)	139.5 (81.0–237.7)*	0.001
sTie-2 (pg/mL)	12.4 (10.2–14.6)	13.6 (11.7–15.2)	11.8 (10.1–14.4)*	12.4 (10.2–14.3)*	0.011

*Note*: Data expressed as median (interquartile range).

* *P*<0.05 compared with normoalbuminuria group (Chi-Square test in categorical variables, Mann-Whitney U test in continuous variables).

** *P*<0.0001 compared with normoalbuminuria group (Chi-Square test in categorical variables, Mann-Whitney U test in continuous variables).

*Abbreviations*: Ang-1, angiopoietin-1; Ang-2, angiopoietin-2; VEGF, vascular endothelial growth factor; sTie-2, soluble Tie-2 receptor.

We further analyzed the relationship between angiotrophic growth factors and eGFR. Linear regression analysis revealed an inverse correlation between Ang-2 levels and eGFR (*r* = -0.245, *P*<0.0001). The endogenous Ang-2 antagonist, sTie-2, was positively correlated with eGFR (*r* = 0.186, *P*<0.0001). Additional analyses of Ang-1 and VEGF only revealed weak association with eGFR (*P* = 0.031 for Ang-1, *P* = 0.182 for VEGF).

### Plasma Ang-2 Associated with Markers of Systemic Microinflammation

Since Ang-2 sensitizes endothelial cells and has a crucial role in the induction of inflammation [10], markers representative of microinflammation were assessed. In the measurements of hsCRP, 78.8% and 56.7% of our patients were >0.05 mg/dL and >0.1 mg/dL respectively. We confirmed the association between the plasma levels of Ang-2 and hsCRP (*r* = 0.114, 95% CI 0.018–0.208, *P* = 0.020, Fig. 1B). Blood uric acid and ferritin level were also positively correlated with plasma levels of Ang-2 (*r* = 0.171, 95% CI 0.076–0.263, *P* = 0.001 for uric acid, and *r* = 0.129, 95% CI 0.033–0.222, *P* = 0.009 for ferritin). On the contrary, the levels of serum albumin and hemoglobin showed an inverse correlation with Ang-2 (*r* = -0.336, 95% CI -0.412– -0.248, *P*<0.0001, Fig. 1C; *r* = -0.322, 95% CI -0.406– -0.233, *P*<0.0001). Among all patients, there were 10.8% with albumin <4 g/dL and 39.1% with hemoglobin <11 g/dL.

### Plasma Ang-2 Associated with Albuminuria Independently

To delineate the association between Ang-2 and ACR, linear regression analyses adjusted for potential confounding factors was performed (Table 3). ACR was log-transformed and treated as the outcome variable. Univariate regression analysis revealed significant association between Ang-2 and ACR (*P*<0.001). After multivariate adjustment, Ang-2 still revealed independent association with ACR (*P* = 0.026). Age, diabetes, mean systolic blood pressure, eGFR and medication in use were further selected in the stepwise regression analysis. We confirmed the independent association between plasma levels of Ang-2 and ACR (*P* = 0.025; with 1 pg/mL increment of Ang-2 increased ACR by 1 mg/g after exponentiation).

**Table 3:** Multivariate-adjusted linear regression analyses of albumin-creatinine ratio and angiopoietin-2^a^.

	Albuminuria (ACR)		
	Regression Coefficient β (10^−4^)	*P*	95% Confidence Interval
**Univariate**			
Ang-2	2.292	<0.001	1.452–3.132
**Multivariate**			
Model 1^b^	2.354	<0.001	1.518–3.191
Model 2^c^	0.921	0.026	0.113–1.730
Model 3^d^	0.902	0.025	0.113–1.692

Note:

a Albuminuria (ACR) was natural logarithm transformed.

b Model 1: Ang-2+age+gender.

c Model 2: Model 1+traditional risk (hypertension, diabetes, dyslipidemia, mean brachial SBP, eGFR) +nontraditional risk (Calcium phosphate product, hemoglobin, high sensitive C reactive protein, medication including ACE inhibitor, ARB, statin, calcium channel blocker, β-blocker, pentoxifylline).

d Model 3: stepwise regression method for variables in model 2.

Abbreviations: ACR, urine albumin-creatinine ratio; SBP, systolic blood pressure; ACE inhibitor, angiotensin-converting enzyme inhibitor; ARB, angiotensin II receptor blocker.

## Discussion

This is the first study that demonstrates the association of plasma Ang-2 with albuminuria and microinflammation in patients with CKD stages 3 to 5. Among the angiogenic growth factors, only Ang-2 shows the positive correlation with albuminuria and hsCRP. We also report inverse correlation between eGFR and plasma Ang-2 levels in moderate to severe CKD patients.

We demonstrated the independent association of plasma Ang-2 with albuminuria in CKD patients. The magnitude of albuminuria is associated with not only increased risk of CVD in general population, but also mortality and ESRD in diverse CKD populations [6], [25]. Several hypotheses have been postulated in translating albuminuria to high cardiovascular risk in CKD [6]. However, the exact mechanism is still obscure. David S et al have reported that increased circulating Ang-2 is associated with CVD and mortality in CKD and dialysis patients [20], [21], [22]. In our CKD patients, plasma levels of Ang-2 were still associated with higher albuminuria after we adjusted for possible confounders, including blood pressure and eGFR. The association between plasma Ang-2 levels and albuminuria might suggest the role of Ang-2 in linking CVD in CKD. It is recognized that CVD increases since the early CKD stage without markedly increased uremic toxins or asymmetric dimethylarginine (ADMA) [2], [26], [27], [28]. Although we cannot demonstrate the causal relationship in this observational study, these observations suggest the crucial role of endothelial damage in the pathogenesis.

We also found the positive correlation between plasma Ang-2 and inflammatory markers in our CKD patients. CKD is recognized as a disease with persistent and low-grade inflammation. Altered cytokine milieu and acute-phase protein, including interleukin-6, TNF-α, and CRP may directly mediate the initiation and progression of CKD complications [9]. Among them, CRP truly reflects a status of microinflammation and increased cardiovascular events [7]. Nevertheless, the functional role of CRP is still an issue of dilemma [29], [30], [31], [32], [33]. In acute inflammation, CRP responds rapidly with wide dynamic range, but direct toxic effect does not appear. In addition to the endothelial effect, Ang-2 has complex effects on inflammatory responses [10], [34]. Noteworthily, there was no correlation between hsCRP and albuminuria in our CKD patients, which might suggest the more promising role of Ang-2 in the endothelial damage and inflammatory induction in CKD.

The mechanism linking endothelial dysfunction, albuminuria and CVD is that endothelial dysfunction leads to increased vascular permeability and glomerular albumin leakage [1]. Increased endothelial Ang-2 secretion is stimulated by exogenous stimuli such as hypoxia, angiotensin II, TNF-α, and reactive oxygen species (ROS) [35]; all of them are common features of CKD and glomerular albuminuria. Mice with podocyte-specific expression of Ang-2 displayed endothelial cell apoptosis and albuminuria [36]. Sustained increases in plasma Ang-2 by conditional expression in liver promoted the remodeling of coronary arteries and reduced the density of endocardial vessels [37]. It is reasonable to propose that endothelial damage in general and glomerular vasculature may lead to endothelial Ang-2 secretion in CKD patients. Elevated Ang-2 might further increase glomerular albuminuria through endothelial damage. However, it is still not clear whether the plasma Ang-2 levels are good indicators of tissue effects in CKD.

There were several limitations in this cross-sectional study. We could only demonstrated the association between Ang-2 and symbolized markers (of microinflammation and vascular injury). In proposing that Ang-2 connects albuminuria to cardiovascular event, we need to investigate the relationship between albuminuria and Ang-2 in early-stage CKD and control group with endothelial dysfunction (such as hypertension or diabetes mellitus). Prospective studies based on Ang-2 stratification are necessary to study the renal and cardiovascular prognosis in CKD. Besides, the mechanisms attributable to increased systemic Ang-2 are not clearly recognized. A delicate animal model is necessary to investigate the exact origin and the causal relationship between Ang-2 and albuminuria in CKD.

In conclusion, plasma Ang-2 was associated with albuminuria and microinflammation in patients with CKD stage 3 to 5. Ang-2 might be a novel non-traditional cardiovascular risk factor in CKD patients. We need further study to identify its role in translating the systemic cardiovascular effect of albuminuria.
